# Mortality during the SARS-CoV-2 Pandemic: A Comparative Analysis between Lombardy in Italy and Israel

**DOI:** 10.3390/jcm13164766

**Published:** 2024-08-13

**Authors:** Ronza Najjar-Debbiny, Alessandro Nobili, Pier Mannuccio Mannucci, Ofra Barnett-Griness, Walid Saliba, Yochai Adir, Alessia Antonella Galbussera, Mauro Tettamanti, Barbara D’Avanzo, Sergio Harari

**Affiliations:** 1 Infection Control and Prevention Unit, Lady Davis Carmel Medical Center, Haifa 3436212, Israel; 2 Ruth and Bruce Rappaport Faculty of Medicine, Technion-Israel Institute of Technology, Haifa 3436212, Israel; salibuss@yahoo.com (W.S.); adir-sh@zahav.net.il (Y.A.); 3 Department of Health Policy, Istituto di Ricerche Farmacologiche Mario Negri IRCCS, 20156 Milan, Italy; alessandro.nobili@marionegri.it (A.N.); alessia.galbussera@marionegri.it (A.A.G.); mauro.tettamanti@marionegri.it (M.T.); barbara.davanzo@marionegri.it (B.D.); 4 Depart Fondazione IRCCS Ca’ Granda Ospedale Maggiore Policlinico, Angelo Bianchi Bonomi Hemophilia and Thrombosis Center, 20122 Milan, Italy; piermannuccio.mannucci@policlinico.mi.it; 5 Department of Community Medicine and Epidemiology, Lady Davis Carmel Medical Center, Haifa 3436212, Israel; barnett_ofra@clalit.org.il; 6 Statistical Unit, Lady Davis Carmel Medical Center, Haifa 3436212, Israel; 7 Translational Epidemiology Unit and Research Authority, Lady Davis Carmel Medical Center, Haifa 3436212, Israel; 8 Pulmonology Unit, Lady Davis Carmel Medical Center, Haifa 3436212, Israel; 9 Department of Clinical Sciences and Community Health, Università di Milano, 20123 Milan, Italy; sergio@sergioharari.it; 10 Division of Internal Medicine, Multimedica IRCSS, 20123 Milano, Italy

**Keywords:** SARS-CoV-2, Lombardy, Israel, mortality, vaccine

## Abstract

**Background:** This retrospective study contrasts the impact of the SARS-CoV-2 pandemic in Lombardy (Italy) and Israel, focusing on mortality, healthcare response, public health measures, and demographics. **Methods:** We analyzed SARS-CoV-2 data from Lombardy and Israel covering four viral waves. Data included infection rates, hospitalizations, and mortality. In Lombardy, healthcare data were collected from the administrative database of the Lombardy Welfare Directorate; in Israel, they were collected from Clalit Health Services and the Israeli Ministry of Health’s COVID-19 database. Statistical analyses compared trends in infection rates, demographics, and mortality rates across the four viral waves by using logistic and linear regression models and adjusting for age, sex, and comorbidities. **Results:** Lombardy exhibited significantly higher SARS-CoV-2 infections and COVID-19 hospitalization rates during the first wave than Israel, with 71,558 cases over a population sample of ~10 million versus 5741 over a population sample of ~4.7 million in Israel. The majority of cases in Israel were managed at home, with 18 cases only (0.3%) requiring intensive care unit (ICU) hospitalization during the first wave, compared to 4104 (5.7%) cases in Lombardy. Israel’s vaccination campaign began earlier, so that by the fourth wave, 439,545 (42.2%) people in Israel were fully vaccinated with three doses, compared to 214,542 (22.9%) in Lombardy. Mortality decreased over time in both sites, dropping from 103 cases (1.8%) to 1550 (0.1%) in Israel and from 13,372 (18.7%) to 4388 (0.3%) in Lombardy. **Conclusions:** Early public health interventions and vaccination were crucial in managing the SARS-CoV-2 impact.

## 1. Introduction

The emergence of the coronavirus disease 2019 (COVID-19), caused by the novel severe acute respiratory syndrome coronavirus 2 (SARS-CoV-2), evolved into a global health crisis [1]. Various measures were implemented worldwide to mitigate mortality rates, which exhibited significant disparities among countries [2]. These variations were attributed to a multitude of factors, including patient demographics, with older age and underlying comorbidities contributing to higher mortality, together with exposure to environmental risk factors, as well as the strain on healthcare facilities and personnel [3]. Consequently, health authorities worldwide grappled with strategies aimed at containing the spread of SARS-CoV-2, primarily through social distancing to flatten and elongate the epidemic curve. In addition, some countries explored the concept of herd immunity by adopting relaxed control measures and thus allowing natural infection to circulate freely in order to establish widespread immunity within the population [4]. In 2021, the introduction of COVID-19 vaccination represented a key milestone in the global efforts to lower the disease impact [5,6].

Israel, a compact nation with a population slightly exceeding 9 million and population density of 421.9/km^2^, employed several strategies to curb the spread of COVID-19 prior to the advent of vaccines. These measures included social distancing, periods of lockdown, travel restrictions, and the implementation of contact tracing through mobile phone technology to ensure isolation of confirmed cases and their close contacts. Furthermore, ambulatory services at healthcare facilities were scaled back, and all medical personnel were redirected towards the care of COVID-19 cases [7].

On the other hand, Lombardy, the largest and most densely populated northern region in Italy, with a population of 10 million people, similar in size to Israel, and a population density of 419.9/km^2^, close to that of Israel, was the first, after China, to bear the brunt of the first wave of the SARS-CoV-2 pandemic in winter–early spring of 2020. Strategies to lower this effect during the first wave included the enforcement of a strict and generalized lockdown that lasted from early March to the end of May 2020. There was also the expansion of intensive care and medical units dedicated to infected cases. The management of affected cases was similar at both sites and based upon the knowledge and recommendation available in the literature at the time of each wave. 

In this study, we sought to compare SARS-CoV-2 pandemic waves in Israel and Lombardy regarding relevant public health data such as number and demography of objectively infected cases, sites of their management, as well as key events such as deaths and hospitalizations, using the population-wide administrative database implemented by the Lombardy Welfare directorate in Italy and the Clalit Health Services database, the largest healthcare provider in Israel.

## 2. Methods

The study was approved by the institutional review board of Lady Davis Medical Centre and the Data Utilization committee of Clalit Health Services (CHS). Owing to the retrospective nature of the study, a waiver of informed consent was granted by the institutional review board. The current study followed the Strengthening the Reporting of Observational Studies in Epidemiology (STROBE) reporting guideline. 

According to Italian law, studies using anonymous data from administrative databases that do not involve direct access to individual patient data need no approval nor notification by an ethics committee/institutional review board and informed patient consent is not required. All data were managed according to Italian law on privacy.

## 3. Study Design

All SARS-CoV-2-positive cases were evaluated across four viral wave time frames: the first was from 1 February until 31 May 2020, the second was from 1 October to 31 December 2020, the third was from 1 February 2021 to 31 May 2021, and the fourth was from 1 December 2021 until 28 February 2022. These time frames were used as a censoring time for the purposes of this study. Cases were considered positive for SARS-CoV-2 when they tested positive by polymerase chain reaction of nasopharyngeal swabs or by positive antigen test when the latter became available. However, in Lombardy, particularly during the first wave, swabs were scarcely accessible outside hospitals, so that the numbers of affected cases managed at home is underestimated. Reinfections were considered when there was a positive test at least 3 months apart from the previous one. Furthermore, data were collected on positive cases, ages of positive cases, number of vaccine doses, the number of drugs consumed in the prior year by the cases, and the drug-derived complexity index (DDCI), used as population proxy for medical complexity [8]. Also, data were collected on the number of SARS-CoV-2 positive cases who died in the forementioned four waves within 28 days from the first positive swab, with information on their age and treatment settings.

### 3.1. Source of Data

#### 3.1.1. Lombardy

The administrative database that collects healthcare data for all residents of the region is regularly updated by the Welfare directorate in order to obtain and record information for administrative, claims, and public health purposes on the use of healthcare facilities by all residents of the region irrespective of age, gender, ethnicity, income, and demographic characteristics. Italian investigators had access to data since the year 2020 on the basis of a research agreement between the Welfare directorate and the Mario Negri Institute of Pharmacological Research, which yielded previous publications on COVID-19 to which we refer for more details on the database and its content [9,10]. In brief, the database section regarding hospital and emergency room attendances supplies admission and discharge dates, deaths or site of discharge, and main diagnoses. All the cases who tested positive for SARS-CoV-2 in the region were included in the database. The drug database section supplies the name, anatomic therapeutic classification (ATC) code, and dispensation date of drugs approved and reimbursed by the National Health Service (NHS), but no information is available for out-of-pocket medications nor those dispensed during hospitalizations or in nursing homes. Data on vaccinations are provided separately from the drug database.

#### 3.1.2. Israel

This study is based on data from two sources: the Clalit Health Services (CHS) database and the Israeli Ministry of Health (MOH) COVID-19 database. We gained access to these databases after authorization by the Data Utilization committee of Clalit Health Services (CHS). Healthcare coverage in Israel is mandatory, according to the National Health Insurance Law (1995), and is provided by four groups akin to not-for-profit health maintenance organizations (HMOs), which are responsible for providing a broad package of benefits stipulated by the government. The four HMOs are both healthcare insurers and providers; therefore, they finance and supply health services. Membership in a specific HMO is voluntary and members can freely switch to another HMO. All members of the different HMOs have a similar health insurance plan and similar access to health services. CHS provides inclusive healthcare for more than half of the Israeli population (~4.7 million) and maintains a database that receives information from multiple sources, including records of primary care physicians, community specialty clinics, hospitalizations, laboratories, and pharmacies. A registry of chronic disease diagnoses is compiled from these data sources. The COVID-19 database is maintained by the Israeli Ministry of Health (MOH) and contains data on vaccination, SARS-CoV-2 polymerase chain reaction (PCR) and antigen tests results, and hospitalizations for COVID-19, including information related to specific departments. The collected data are transferred daily to healthcare providers. Several high-quality studies related to COVID-19 have been conducted based on integrated data from these two databases [11,12,13].

All data necessary for the purpose of this study are presented in aggregation in this manuscript. Due to the data privacy regulations of Clalit Health Services and to the data utilization institutional approval for this study, the individual level data cannot be shared. The same conditions apply to the Lombardy region’s data.

## 4. Data Analysis

The main characteristics of the cases are presented as means and standard deviations or medians and interquartile ranges for continuous variables (i.e., age and drug number) and numbers with percentages for categorical variables (all the remaining ones). Differences among the waves in sex composition, age groups, place of management, and death proportion were tested with logistic regression and by reporting frequencies or odds ratios (ORs) and 95% confidence intervals (95%Cis); the same was carried out to test differences among patients who died in the different waves for sex and age groups. For continuous variables (age, number of drugs, and drug-derived complexity index (DDCI)), a linear regression model was fitted and differences between the waves were reported as mean changes and 95%Ci. All the fitted models were adjusted by sex, age, and DDCI obtained as a proxy of comorbidities and medical complexity, except for the number of drugs, where only sex and age were used for adjustment. Statistical analyses were performed using SAS software, SAS Institute Inc., Cary, NC, USA.

## 5. Results

The number of infected patients varied between waves both in Israel and Lombardy, exhibiting an increasing trend over the course of the waves. Notably, during the first wave, the numbers were considerably higher in Lombardy compared to Israel (71,558 vs. 5741, respectively, 7/1000 and 0.5/1000) (Table 1). The mean age of infected cases in Lombardy was higher than in Israel, with a notable gap during the first wave—60.8 (SD = 18.4) in Lombardy compared to 40.3 (SD = 22.9) in Israel. In Israel, the majority of the cases across all waves were under the age of 50, whereas in Lombardy, during the first wave, most patients were aged 50 and above (Table 1). In Israel, most cases were managed at home without the need for hospitalization across all waves, and only 18 cases (0.3%) required intensive care unit (ICU) hospitalization during the first wave, in contrast to 4104 (5.7%) cases in Lombardy (Table 1).

Concerning the number of drugs, which was used as a proxy of comorbidities and clinical complexity in positive patients, in the first wave, the number of drugs was slightly higher in Lombardy patients, with a mean of 4.5 and standard deviation of 4.8, as opposed to 4.4 (SD = 4.6) in Israeli patients. An opposite relation is seen during the subsequent waves (Table 1). The number of drugs dispensed one year before SARS-CoV-2 positivity is higher along all waves in the Israeli group in patients who died with COVID-19, as seen in Table 2.

The vaccination campaign commenced much earlier in Israel, as shown in Table 1. During the second wave, 714 (0.9%) people in Israel received one dose, while in Lombardy, only 4 received a single dose. In the fourth wave, 439,545 (42.2%) people in Israel were fully vaccinated with three doses, and 36,707 (3.5%) received a fourth dose. In contrast, in Lombardy, only 214,542 (22.9%) were fully vaccinated with three doses during the fourth wave, but none were vaccinated with a fourth dose.

The mortality rate declined across the viral waves, from 103 (1.8%) to 1550 (0.1%) in Israel and from 13,372 (18.7%) to 4388 (0.3%) in Lombardy. The standardized mortality ratio, using Lombardy as the reference population, was 0.2 in the first wave but became 0.5 by the fourth wave, as shown in Table 1.

Table 2 presents the main characteristics of SARS-CoV-2-positive patients who died across all waves. It reveals that the mean age of patients who died ranged from 75.7 (SD = 15.2) to 81.7 (SD = 12.6) in Israel, while in Lombardy, it ranged between 78.5 (SD = 10.1) and 81.6 (SD = 11.8). In both countries, the majority of cases who died were aged 70 years and older across all waves.

## 6. Discussion

This study compared SARS-CoV-2 infection trends over four pandemic waves in Lombardy and Israel, using healthcare database analysis. The countries are both high-income countries and both provide coverage of healthcare to all citizens, albeit with different organizations. Lombardy experienced higher initial infection numbers, yet both sites achieved significant reductions in mortality over time.

The first COVID-19 cases were detected in Israel at the end of February [14], about three weeks later than in Lombardy. As mentioned, Lombardy has some common characteristics with Israel, such as universal healthcare coverage and similar population size and density. However, the median age of the Israel population is much lower than in Lombardy, indicating a higher proportion of young citizens. The weather conditions in Israel are warmer than in Lombardy [14]; the latter has a higher burden of ambient air pollution. During the first SARS-CoV-2 wave, Lombardy had a larger proportion of older patients, a higher level of comorbidity and clinical complexity according to the DDCI score, and higher rates of hospitalization, particularly in intensive care. Concerning the number of drugs dispensed one year before SARS-CoV-2 positivity, in the first wave, the number of drugs was slightly higher in Lombardy patients. An opposite relation was seen during the subsequent waves. Israel’s faster response to the pandemic and more comprehensive vaccination campaign likely contributed to the more pronounced mortality decline. The observed differences in mortality rates between the two sites persisted, particularly during the first wave, even after adjusting for age differences by means of the standardized mortality rate. None of the forementioned characteristics of each country (population size, climate, age distribution, comorbidities, etc.) can fully explain the extreme differences in COVID-related mortality rates, which were nearly 10 times higher in Lombardy than in Israel in the first wave [14,15].

This difference in the number of deaths, as recorded in this study, is supported by a recent comprehensive analysis in the context of a report of the 2021 Global Burden of Disease (GBD) collaboration on the demographic changes that occurred during the 2020–2021 SARS-CoV-2 pandemic period [2]. On the whole, Israel total deaths in 2021 were 50.100 and the calculated excess deaths due to COVID-19 were 2000 in 2020 and 3000 in 2021. The GBD analysis does not provide data for Lombardy but provides data for the whole country of Italy. The differences with Israel in the corresponding periods are striking: total deaths in 2021 were 699.000 in Italy, and calculated excess deaths due to COVID-19 were 98,000 in 2020 and 63,000 in 2021. 

This discrepancy could be linked to several factors. The first non-imported COVID-19 case was documented in Lombardy in late February 2020 [15]. The initial case of domestic transmission in Israel was reported in early March [14], by which time the availability of PCR testing was high. This provided Israel with additional preparation time for the impending pandemic [14] and allowed for the effective implementation of the “flattening the curve” strategy, which has been demonstrated to be effective in numerous models. For instance, according to Walkowiak et al. [16], delaying the peak of the wave reduced excess deaths by 1.79% for each week. Israel adopted this strategy during the first wave by imposing lockdowns and isolating exposed individuals through phone tracing; the latter measure was not utilized in Lombardy during the same timeframe. Moreover, in February 2021, Israel launched an extensive vaccination campaign, achieving high vaccination rates early in the pandemic with the aim of reaching herd immunity quickly. It is likely that other factors need to be taken in account to explain the differences. One is the role of ambient air pollution, considering that Lombardy is located in the Po valley, one of the most polluted areas of Europe [17,18]. We must also mention that the spread and impact of the SARS CoV-2 infection and COVID-19 were not homogenous throughout Italy: during the first wave, Bergamo, the Lombardy town most heavily affected by the pandemic, experienced an excess of mortality of 586%, while Trieste, a seaside city of the north of Italy, in the Friuli-Venezia Giulia eastern region and 370 Km away from Bergamo, had an excess mortality of only 15% [19]. We believe that the high burden on the healthcare system in Lombardy during the first wave also affected the management of subsequent waves. 

A systematic review by Girum et al., encompassing 25 studies, consistently underscored the advantages of social distancing, stay-at-home orders, travel restrictions, and lockdown measures, all of which were implemented by Israel early during the first three waves [20]. These strategies effectively reduced contact rates, daily infection growth, and the median number of infections, thereby controlling the spread of the epidemic. The proactive implementation of these measures from the beginning of the pandemic in Israel resulted in a lower number of positive cases across all waves compared to Lombardy, likely explaining, at least in part, the differences in mortality rates observed. Additionally, the excess mortality in Lombardy during the first wave may be attributed to the overwhelming of the healthcare system by the new viral diseases [21,22].

This study has several limitations. First, the limitations on the release of some data imposed by the institutional review board and Clalit Health Services in Israel and by the regional Welfare Department in Lombardy made it impossible to perform a more advanced statistical analysis of the case material of the two cohorts, such as, for instance, propensity score matching. Also, no data were available for treatments provided across all waves in both sites, although we believe both countries adhered to standard of care treatments relevant to each wave based on the international literature. Moreover, we acknowledge that the time frames of the viral waves are those established by the Italian National Institute of Health because Italy was the first country in the world to be affected after China. However, we believe that the difference in time frames from those of the WHO are small and should not impinge upon the impact and significance of the findings. Despite these limitations, we believe that the data are comprehensive enough, considering also the many high-quality studies that have been conducted and published based on both databases [9,10]. Finally, being aware that in both countries the number of positive cases was highly dependent on the number of tests conducted, we recognize that this might have influenced, to some extent, the number of asymptomatic patients treated at home, particularly in Lombardy, but this likely had a smaller impact on the number of hospitalized patients.

In conclusion, this study provides an analysis of the SARS-CoV-2 infection trends across four pandemic waves in two sites (Lombardy and Israel) with similar population size and universal healthcare coverage. The findings highlight the pivotal role of early and aggressive public health interventions, the impact of demographic factors, and the significance of rapid vaccination rollouts in controlling the spread of the virus and, ultimately, reducing mortality rates.

## Figures and Tables

**Table 1:** Main characteristics of SARS-CoV-2 infection cases throughout the four waves.

Variables	First Wave Lombardy	First Wave Israel	Second Wave Lombardy	Second Wave Israel	Third Wave Lombardy	Third Wave Israel	Fourth Wave Lombardy	Fourth Wave Israel
Total cases of SARS-CoV-2 positivity	N = 71,558	N = 5741	N = 359,173	N = 80,820	N = 289,603	N = 91,626	N = 1,355,674	N = 1,041,350
Proportion of cases of SARS-CoV-2 positivity per 1000 inhabitants	*7.2*	*1.2*	*35.9*	*16.8*	*29.0*	19.1	*135.6*	*217*
Sex, females, N (%) adjusted OR * (95% CI)	34,556 (48.3) 1	2709 (47.2) 1	182,432 (50.8) 1.13 (1.11; 1.15)	42,941 (53.1) 1.30 (1.23; 1.37)	144,117 (49.8) 1.09 (1.07; 1.10)	49,624 (54.2) 1.41 (1.34; 1.49)	706,893 (52.1) 1.21 (1.19; 1.23)	571,413 (54.9) 1.43 (1.36; 1.51)
Age, mean (SD) adjusted difference ** (95% CI)	60.8 (18.4) 0	40.3 (22.9)	44.7 (20.9) −9.15 (−9.30; −9.00)	36.1 (21.9) −3.57 (−4.11; −3.04)	44.4 (21.9) −9.39 (−9.54; −9.23)	27.0 (19.6) −11.50 (−12.03; −10.97)	37.2 (21.0) −14.47 (−14.61; −14.32)	30.0 (21.4) −9.07 (−9.59; −8.55)
Age groups, N (%)								
<=29	3944 (5.5)	2239 (39.0)	94,709 (26.4)	35,094 (43.4)	80,237 (27.7)	55,145 (60.2)	521,903 (38.5)	544,216 (52.3)
30–49	15,477 (21.6)	1549 (27.0)	111,993 (31.2)	23,845 (29.5)	84,747 (29.3)	23,908 (26.1)	435,225 (32.1)	302,228 (29.0)
50–69	26,581 (37.2)	1227 (21.4)	105,650 (29.4)	14,976 (18.5)	84,137 (29.1)	9306 (10.2)	301,927 (22.3)	139,110 (13.4)
70+	25,556 (35.7)	726 (12.6)	46,821 (13.0)	6905 (8.5)	40,482 (14.0)	3262 (3.6)	96,619 (7.1)	55,783 (5.4)
Cases managed, N (%)								
– At home	28,059 (39.2)	4508 (78.5)	319,286 (88.9)	76,002 (94.0)	253,003 (87.4)	87,931 (96.0)	1,333,460 (98.4)	1,032,213 (99.1)
– Hospitalized	39,395 (55.1)	1215 (21.2)	37,375 (10.4)	4248 (5.3)	33,624 (11.6)	3353 (3.7)	21,303 (1.6)	8663 (0.8)
– ICU	4104 (5.7)	18 (0.3)	2512 (0.7)	570 (0.7)	2976 (1.0)	342 (0.4)	911 (0.1)	474 (0.0)
Number of drugs dispensed one year before SARS-CoV-2 positivity, mean (SD) adjusted difference ** (95% CI)	4.5 (4.8) 0	4.4 (4.6) 0	2.4 (3.4) −1.09 (−1.11; −1.07)	3.9 (4.3) −0.15 (−0.23; −0.06)	2.1 (3.2) −1.35 (−1.37; −1.33)	2.6 (3.4) −0.58 (−0.67; −0.50)	1.5 (2.5) −1.54 (−1.56; −1.52)	3.3 (3.7) −0.23 (−0.31; −0.14)
DDCI (drug-derived complexity index), mean (SD) adjusted difference ** (95% CI)	2.5 (3.5) 0	0.7 (1.9) 0	1.0 (2.3) −0.78 (−0.79; −0.76)	0.6 (1.7) −0.05 (−0.09; −0.01)	1.0 (2.3) −0.78 (−0.79; −0.76)	0.3 (1.3) −0.08 (−0.11; −0.04)	0.6 (1.7) −0.92 (−0.93; −0.91)	0.5 (1.5) −0.04 (−0.08; −0.00)
Died with SARS-CoV-2 positivity, N (%) adjusted OR * (95% CI)	13,372 (18.7) 1	103 (1.8) 1	8487 (2.4) 0.22 (0.21; 0.22)	741 (0.9) 0.81 (0.64; 1.02)	6158 (2.1) 0.18 (0.18; 0.19)	360 (0.4%) 0.83 (0.65; 1.06)	4388 (0.3) 0.05 (0.05; 0.05)	1550 (0.1) 0.20 (0.16; 0.25)
Standardized mortality ratio (SMR) *** (95% CI)	0.23 (0.19; 0.28)		0.56 (0.52; 0.6)		0.67 (0.6; 0.74)		0.53 (0.5; 0.55)	
Vaccinated before SARS-CoV-2 positivity, N (%)	-	-	4 (0.0)	714 (0.9)	13,819 (4.8)	18,109 (19.8)	936,679 (69.1)	714,801 (68.6)
– One dose	-	-	4	714 (0.9)	10,430 (3.6)	13,978 (15.3)	63,798 (4.7)	38,504 (3.7)
– Two doses	-	-	-	-	3389 (0.01)	4131 (4.5)	658,339 (48.9)	200,045 (19.2)
– Three doses	-	-	-	-	-	-	214,542 (15.8)	439,545 (42.2)
– Four doses								36,707 (3.5)

**Legend**: **Waves: the first wave** from 20 February until 31 May 2020; **the second** from September 15 to 15 December 2020; **the third** from 16 December 2020 to 15 June 2021; and **the fourth** from 1 December 2021 until February 2022, when administrative data were last censored and made available to us. * Odds ratios/differences were adjusted for age sex and DDCI (number of drugs was not corrected for DDCI). ** Logistic regression for sex and death, linear regression for age, number of drugs, and DDCI. *** SMR using Lombardy as the standard population.

**Table 2:** Main characteristics of patients who died with SARS-CoV-2 positivity throughout the four waves.

Variables	First Wave Lombardy	First Wave Israel	Second Wave Lombardy	Second Wave Israel	Third Wave Lombardy	Third Wave Israel	Fourth Wave Lombardy	Fourth Wave Israel
Cases who died with SARS-CoV-2 positivity	N = 13,372	N = 103	N = 8487	N = 741	N = 6158	N = 360	N = 4388	N = 1550
Sex, females, N (%) adjusted OR * (95% CI)	4544 (34.0) 1	50 (48.5) 1	3412 (40.2) 1.18 (1.11; 1.25)	321 (43.3) 0.83 (0.55; 1.26)	2468 (40.1) 1.19 (1.12; 1.27)	178 (49.4) 1.15 (0.74; 1.80)	1970 (44.9) 1.42 (1.32; 1.52)	746 (48.1) 0.95 (0.63; 1.42)
Age, mean (SD) adjusted difference ** (95% CI)	78.5 (10.1) 0	80.7 (15.7) 0	81.2 (10.1) 2.38 (2.11; 2.66)	79.2 (12.5) −1.38 (−4.05; 1.29)	80.7 (9.9) 1.87 (1.57; 2.17)	75.7 (15.2) −4.97 (−7.81; −2.13)	81.6 (11.8) 2.65 (2.31; 2.99)	81.7 (12.6) 0.93 (−1.66; 3.52)
Age groups, N (%)								
<=29	5 (0.0)	2 (1.9)	13 (0.2)	2 (0.3)	3 (0.1)	8 (2.2)	22 (0.5)	8 (0.5)
30–49	152 (1.1)	2 (1.9)	64 (0.8)	17 (2.3)	30 (0.5)	13 (3.6)	64 (1.5)	20 (1.3)
50–69	2082 (15.6)	16 (15.5)	871 (10.3)	116 (15.7)	741 (12.0)	77 (21.4)	458 (10.4)	185 (11.9)
70+	11,133 (83.3)	83 (80.6)	7539 (88.8)	606 (81.8)	5384 (87.4)	262 (72.8)	3844 (87.6)	1337 (86.3)
Number of drugs dispensed one year before SARS-CoV-2 positivity, mean (SD) adjusted difference * (95% CI)	8.2 (5.3) 0	12.0 (4.9) 0	8.4 (5.2) 0.04 (−0.11; 0.18)	11.9 (5.3) 0.03 (−1.13; 1.19)	8.1 (5.1) −0.22 (−0.37; −0.06)	11.2 (5.6) −0.55 (−1.79; 0.68)	7.6 (5.1) −0.78 (−0.96; −0.60)	12.6 (5.9) 0.58 (−0.55; 1.70)
DDCI (drug-derived complexity index), mean (SD) adjusted difference * (95% CI)	5.1 (4.0) 0	3.6 (3.3) 0	5.3 (4.0) 0.02 (−0.09; 0.13)	3.8 (3.4) 0.24 (−0.51; 0.99)	5.4 (4.0) 0.13 (0.01; 0.25)	3.3 (3.3) −0.25 (−1.05; 0.55)	5.2 (4.0) −0.17 (−0.31; −0.04)	4.4 (3.8) 0.87 (0.15; 1.60)
Mortality on total cases with SARS-CoV-2 positivity								
– At home, N of deaths/N of total cases with SARS-CoV-2 positivity (%)	2088/28,059 (7.4)	7/4508 (0.16)	1967/319,286 (0.6)	59/76,002 (0.08)	996/253,003 (0.4)	26/87,931 (0.03)	1750/1,333,460 (0.1)	330/1,032,213 (0.03)
– Hospitalized, N of deaths/N of total cases with SARS-CoV-2 positivity (%)	9527/39,395 (24.2)	92/1215 (7.6)	5617/37,375 (15.0)	509/4248 (12.0)	4285/33,624 (12.7)	268/3353 (8.0)	2371/21,303 (11.1)	1076/8663 (12.4)
– ICU, N of deaths/N of total cases with SARS-CoV-2 positivity (%)	1757/4104 (42.8)	4/18 (22.2)	903/2512 (35.9)	173/570 (30.4)	877/2976 (29.5)	66/342 (19.3)	267/911 (29.3)	144/474 (30.4)

**Legend**: **Waves: the first wave** from 20 February until 31 May 2020; **the second** from 15 September to 15 December 2020; **the third** from 16 December 2020 to 15 June 2021; and **the fourth** from 1 December 2021 until February 2022, when administrative data were last censored and made available to us. * Odds ratios/differences were adjusted for age sex and DDCI (number of drugs was not corrected for DDCI). ** Logistic regression for sex, linear regression for age, number of drugs, and DDCI.

## Data Availability

Only upon permission by the Lombardy Region, Clalit Healthcare Services (CHS), and the Israeli Ministry of Health (MOH).
